# Resveratrol and FGF1 Synergistically Ameliorates Doxorubicin-Induced Cardiotoxicity via Activation of SIRT1-NRF2 Pathway

**DOI:** 10.3390/nu14194017

**Published:** 2022-09-27

**Authors:** Guangping Lu, Qingbo Liu, Ting Gao, Jiahao Li, Jingjing Zhang, Ou Chen, Cong Cao, Min Mao, Mengjie Xiao, Xiaohui Zhang, Jie Wang, Yuanfang Guo, Yufeng Tang, Junlian Gu

**Affiliations:** 1 School of Nursing and Rehabilitation, Cheeloo College of Medicine, Shandong University, Jinan 250012, China; 2 Department of Cardiology at the First Hospital of China Medical University, Shenyang 110016, China; 3 Department of Orthopedic Surgery, The First Affiliated Hospital of Shandong First Medical University, Jinan 250014, China

**Keywords:** doxorubicin, cardiotoxicity, SIRT1, NRF2, oxidative stress

## Abstract

Doxorubicin (DOX) has received attention due to dose-dependent cardiotoxicity through abnormal redox cycling. Native fibroblast growth factor 1 (FGF1) is known for its anti-oxidative benefits in cardiovascular diseases, but possesses a potential tumorigenic risk. Coincidentally, the anti-proliferative properties of resveratrol (RES) have attracted attention as alternatives or auxiliary therapy when combined with other chemotherapeutic drugs. Therefore, the purpose of this study is to explore the therapeutic potential and underlying mechanisms of co-treatment of RES and FGF1 in a DOX-treated model. Here, various cancer cells were applied to determine whether RES could antagonize the oncogenesis effect of FGF1. In addition, C57BL/6J mice and H9c2 cells were used to testify the therapeutic potential of a co-treatment of RES and FGF1 against DOX-induced cardiotoxicity. We found RES could reduce the growth-promoting activity of FGF1. Additionally, the co-treatment of RES and FGF1 exhibits a more powerful cardio-antioxidative capacity in a DOX-treated model. The inhibition of SIRT1/NRF2 abolished RES in combination with FGF1 on cardioprotective action. Further mechanism analysis demonstrated that SIRT1 and NRF2 might form a positive feedback loop to perform the protective effect on DOX-induced cardiotoxicity. These favorable anti-oxidative activities and reduced proliferative properties of the co-treatment of RES and FGF1 provided a promising therapy for anthracycline cardiotoxicity during chemotherapy.

## 1. Introduction

Doxorubicin (DOX) is one of the most important agents for chemotherapeutic treatment of hematologic malignancies and solid tumors [1]. However, its accumulative and dose-dependent multi-organ toxicities, such as cardiotoxicity, hepatotoxicity, nephrotoxicity and gonad toxicity, is the inherent challenge of DOX which limits its application and effectiveness [2,3,4]. Although, it has been suggested that the heart is the premier target of DOX toxicity [5]. Furthermore, heart failure incidences progressively increase in patients who have received an accumulative dose of DOX exceeding 500 mg/m^2^ [6]. Notably, oxidative stress has been identified to play a crucial role in DOX-mediated cardiotoxicity [7]. Thus, developing cardiac-protective agents to increase physiological antioxidant capability and reduce reactive oxygen species (ROS) production might be a crucial strategy to improve DOX-related cardiotoxicity.

Multiple works identified that fibroblast growth factor 1 (FGF1) is elevated by oxidative damage, implying that an increased FGF1 expression is an adaptive response and might be protective under different kinds of stresses. Our previous study revealed that FGF1 displayed therapeutics and favorable effects on maintaining myocardial redox homeostasis and improving cardiac function, especially in the setting of DOX treatment [8,9]. Beyond that, FGF1 is now emerging as possessing various protective effects including but not restricted to promoting angiogenesis [10] and alleviating apoptosis [11] and fibrosis [9] through partly attenuating oxidative stress. These findings support the idea that FGF1 has a great potency in the prevention and treatment of heart injury in response to stresses. However, the long term use of a wild-type FGF1, as a classically known mitogen, may enhance tumorigenic risks and increase the rate of metastasis owing to its strong mitogenic activity, which limits the use of FGF1 in clinic cancer-prone diseases. Thus, it is urgent to find a drug/natural product to antagonize the proliferative activities of FGF1 and further increase its powerful protective effect on the heart.

Resveratrol (RES), a natural phytoalexin present in grapes, peanuts, mulberries and other plants, has received much attention for its anti-cancer properties due to its anti-proliferative and pro-apoptotic effects in multiple cancer cell types [12,13,14,15]. Meanwhile, RES has long been identified as an antioxidant to exert cardioprotective effects, which also could reduce the DOX-related ROS level in cardiomyocytes [16]. A recent study has shown that RES resistance to DOX-induced oxidative stress depends on sirtuin 1 (SIRT1) activation. In addition, the available data clearly suggests that the indirect target of RES is nuclear factor erythroid 2-related factor 2 (NRF2) [17], which is indispensable in response of an antioxidant by up-regulating various antioxidant enzymes [18]. Alavi et al. has demonstrated that RES can reduce the occurrence of tumors by activating SIRT1 or NRF2 [19,20]. Nonetheless, considering that FGF1 induces proliferation and differentiation in a wide variety of cells, which has greatly limited its clinical use. Therefore, in the present study, we expect to further evaluate whether RES antagonizes the anti-proliferation activity of FGF1 and provides much more effective cardiac antioxidant protection against DOX.

To address these issues, the dual cardioprotective and antitumor action of RES in combination with FGF1 was examined in different kinds of cancer cells and a mouse model of DOX-induced cardiotoxicity, respectively. We found that RES obviously decreased the oncogenesis effect of FGF1, and meanwhile a DOX-impaired myocardial structure, oxidative stress, apoptosis and inflammation were significantly reversed by the co-treatment of RES and FGF1. Furthermore, mechanistic studies on DOX-exposed embryonic rat myocardium-derived cells (H9c2) demonstrated that a short hairpin RNA (shRNA) knockdown of either *Sirt1* or *Nrf2* weaken the improved effect of RES/FGF1 on oxidative stress, which might rely on a positive autoregulatory feedback loop between SIRT1 and NRF2. Collectively, this study could provide a greater possibility for considering a combination of RES and FGF1 as a promising strategy in preventing anthracycline-induced heart disease and concomitantly synergizing with chemotherapy to delay cancer cell growth.

## 2. Materials and Methods

### 2.1. Animals and Treatments

Eight-week-old C57BL/6J male mice, acquired from Vital River Laboratories (Beijing, China), were maintained in a specific pathogen-free facility in a controlled environment (22 °C, 12 h shift of the sleep-wake cycle) and given free access to adequate food and tap water. All animals were acclimatized for 1 week before experimentation and divided into five groups (Ctrl, DOX, DOX/RES, DOX/FGF1 and DOX/RES/FGF1) with 6 mice in each group. RES (10 mg/kg/day) and FGF1 (0.5 mg/kg/day) or the same volume of vehicle (saline) was intraperitoneally injected for seven consecutive days. At the end of the RES plus FGF1 treatment, 20 mg/kg DOX or the same volume of vehicle (saline) was given by a single intraperitoneal injection. Cardiac tissues were harvested 24 h after the DOX injection. All mouse protocols and experiments were approved by the Animal Care and Utilization Committee of Shandong University (Shandong, China).

### 2.2. Cell Culture and Treatments

H9c2 were purchased from the American Type Culture Collection (ATCC, Manassas, VA, USA) and were cultivated with high-glucose DMEM (Macgene, Beijing, China) supplemented with 10% fetal bovine serum (Gibco, Grand Island, NY, USA), 100 U/mL of penicillin and 100 μg/mL of streptomycin in a 5% CO_2_ incubator at 37 °C for the following analyses. For study of the down-regulation of *Sirt1* or *Nrf2*, H9c2 cells were pretreated with a bacterial stab of the plasmid shRNA-negative control (NC-shRNA), *Sirt1*-shRNA or *Nrf2*-shRNA which were purchased from GenePharma (Shanghai, China). Following the manufacturer’s instructions, H9c2 cells were transfected with transfection reagent (Obio Technology, Shanghai, China) at 70–90% confluence. The cells were treated with DOX (1 μM, MedChemExpress, Monmouth Junction, NJ, USA) in the presence or absence of RES (20 μM, MedChemExpress), FGF1 (100 ng/mL, obtained from School of Pharmaceutical Sciences at the Wenzhou Medical University) and NRF2 activator sulforaphane (SFN, 10 μM, MedChemExpress) at 37 °C for 24 h.

### 2.3. Cell Viability

The cell proliferation was determined using a Cell Counting Kit-8 (CCK-8) kit (Beyotime Biotechnology, Shanghai, China). In brief, a human hepatocellular carcinoma (HepG2), bladder cancer (5637) and breast cancer (MCF-7) cells were planted at a density of 3 × 10^3^ cells/well in 96-well multiplates, then treated with RES and (or) FGF1. After 24 h, 10 μL of the CCK-8 solution was added to each well and further incubated for 1 h. Then, the absorbance values were detected at a wavelength of 450 nm. The cell viability was calculated using the optical density values.

### 2.4. Measurement of CK, LDH, GSH and MDA Levels

Serum and cardiac tissues were acquired by every group of mice and stored at −80 °C for subsequent analyses. To analyze the cardiac injury, the levels of creatine phosphokinase (CK) and lactate dehydrogenase (LDH) in serum were detected by using a colorimetry kit (Nanjing Jiancheng Biological Engineering Institution, Nanjing, China) and a commercial kit (Solarbio, Beijing, China). To explore the oxidative stress levels, the content of glutathione (GSH) in serum and the malondialdehyde (MDA) levels in cardiac tissues were tested by using the commercial assay kits (Solarbio) according to the instructions, respectively.

### 2.5. Enzyme-Linked Immunosorbent Assay (ELISA)

Concentrations were determined referring to the standard curve, and the cardiac troponin I (cTnI) levels in serum were tested by the ELISA kits (Cat#SEKM-0153, Solarbio) following the manufacturer’s instructions.

### 2.6. Histology and Cellular Staining

The mice hearts were isolated, fixed in 10% formalin and processed via dehydrating, embedding and sectioning. Immunohistochemical (IHC) staining was done with anti-Cleaved caspase-3 (1:500, Cat#9664S, Cell Signaling Technology, Danvers, MA, USA) and anti-SIRT1 (1:200, Cat#8469, Cell Signaling Technology), immunofluorescent (IF) staining was done with anti-NRF2 (1:200, Cat#ab31163, Abcam, Cambridge, UK), anti-heme oxygenase-1 (HO-1, 1:200, Cat#10701-1-AP, Proteintech, Rosemont, IL, USA) and anti-NAD(P)H quinone dehydrogenase 1 (NQO1, 1:50, Cat#sc-32793, Santa Cruz Biotechnology, Dallas, TX, USA) and the anti-Cluster of Differentiation 68 (CD68, 1:200, Cat#28058-1-AP, Proteintech) was performed as described previously [21].

Three-micrometer-thick cardiac tissue sections and MCF-7 cells were fixed in 4% paraformaldehyde for 20 min at 15 to 25 °C and permeabilized with a mixture of 0.1% Triton X-100 and 0.1% sodium citrate. Subsequently, these tissues and cells were incubated with a terminal deoxynucleotidyl transferase reaction mixture in a dark and humid atmosphere at 37 °C for 60 min using an In Situ Cell Death Detection Kit (Roche Diagnostics GmbH, Mannheim, Germany). After the incubation, DAPI (Abcam, Cambridge, MA, USA) was applied for nuclear staining. The apoptotic cells were detected by a fluorescence microscope, and excitation wavelengths in the range of 570–620 nm were used.

The generation of the ROS were applied to evaluate, using dihydroethidium (DHE) staining and 2′,7′-dichlorofluorescin diacetate (DCFH-DA) staining. The pre-treated H9c2 cells were stained with a DHE fluorescence kit (Beyotime Biotechnology) and a DCFH-DA fluorescence kit (Beyotime Biotechnology) according to the respective standard protocols.

### 2.7. Quantitative Real-Time PCR (qRT-PCR)

The total RNA samples were isolated from heart samples with TRIzol reagent (Cwbio, Valencia, CA, USA). A HiFiScript cDNA Synthesis kit (Cwbio) was used for the reverse transcription of RNA. The primers of interleukin-6 (*Il6*), interleukin-1α (*Il1a*), interleukin-6 (*Il1b*), monocyte chemoattractant protein-1 (*Mcp1*), tumor necrosis factor-α (*Tnfa*) and Glyceraldehyde-3-phosphate dehydrogenase (*Gapdh*) were obtained from Sangon Biotech (Shanghai, China). The expression level of every target gene was normalized to GAPDH.

### 2.8. Western Blot Analysis

The protein of either cardiac tissues or H9c2 cells was isolated using a RIPA lysis buffer (Beyotime Biotechnology) supplemented with protease and phosphatase inhibitors (Beyotime Biotechnology) on ice. The protein concentrations were determined using the BCA protein assay kit (Beyotime Biotechnology). The samples mixed with the loading buffer were heated for 5 min at 95 °C and subjected to electrophoresis on 10% SDS-PAGE gel and electrotransferred to a nitrocellulose membrane (GE Healthcare Life Sciences, Beijing, China). After blocking with 5% nonfat milk for 1 h, the primary antibody was incubated at 4 °C overnight: anti-Cleaved caspase-3 (1:1000), anti-BAX (1:1000, Cat#2772, Cell Signaling Technology), anti-B-cell lymphoma-2 (BCL-2, 1:1000, Cat#3498, Cell Signaling Technology), anti-SIRT1 (1:1000), anti-p65 (1:1000, Cat#8242, Cell Signaling Technology), anti-p-p65 (1:1000, Cat#3033, Cell Signaling Technology), anti-IKBα (1:1000, Cat#4814, Cell Signaling Technology), anti-p-IKBα (1:1000, Cat#ab133462, Abcam), anti-catalase (CAT, 1:1000, Cat#sc-271803, Santa Cruz Biotechnology), anti-superoxide dismutase 1 (SOD1, 1:1000, Cat#sc-101523, Santa Cruz Biotechnology), anti-SOD2(1:1000, Cat#sc-137254, Santa Cruz Biotechnology), anti-NRF2 (1:1000), anti-Histone H3 (1:1500, Cat#BF9211, Affinity Biosciences, Cincinnati, OH, USA), anti-HO-1 (1:1000), anti-NQO1 (1:200), anti-Acetyl-p53 (Ac-p53, 1:1000, Cat#2570, Cell Signaling Technology), anti-Ac-FOXO1 (1:1000, Cat#sc-49437, Santa Cruz Biotechnology), anti- Kelch-like ECH-associated protein 1 (Keap1, 1:1000, Cat#8047, Cell Signaling Technology) and anti-GAPDH (1:1000, Cat#GB11002, Servicebio Technology). Then the next day, the secondary antibody was diluted with the blocking solution and incubated for 1 h at room temperature. The probed proteins were visualized using an enhanced chemiluminescence detection kit (Millipore, Billerica, MA, USA). Densitometric analysis was done using Image Quant 4.2 software (Tanon, Shanghai, China).

### 2.9. Statistical Analysis

Data are expressed as mean ± standard deviation (SD). Statistical analysis was calculated using one-way ANOVA and followed by post hoc pairwise comparisons using Tukey’s test with GraphPad Prism. 8.0. *p* < 0.05 was considered statistically significant.

## 3. Results

### 3.1. RES Significantly Antagonizes the Oncogenesis Effect of FGF1

**Figure 1:**
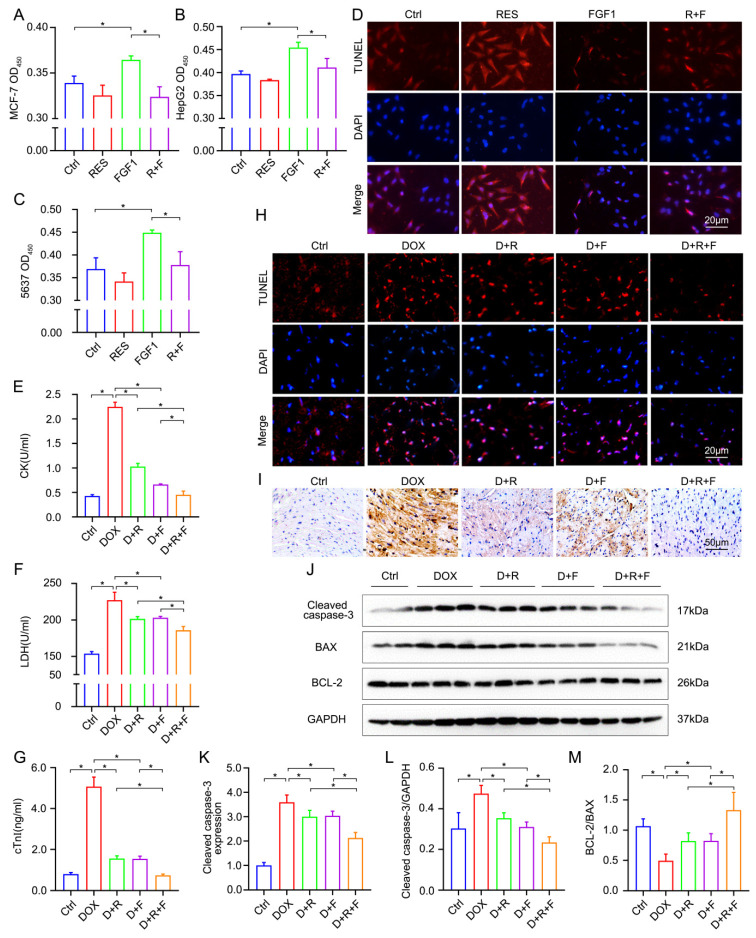
RES and FGF1 co-treatment inhibited the oncogenesis effect of FGF1 as well as alleviated myocardial injury and apoptosis in DOX-related mice. (**A**–**C**) The cell viability was measured in MCF-7, HepG2 and 5637 cells by CCK-8 assay, respectively (*n* = 3). (**D**) The apoptosis of MCF-7 cells was detected by TUNEL staining. (**E**–**G**) The levels of CK, LDH and cTnI were tested in serum (*n* = 6). (**H**) Visualization of cardiac apoptosis by TUNEL staining in cardiac tissues. (**I**,**K**) Representative immunohistochemical (IHC) staining images and statistical results of Cleaved caspases-3 (*n* = 6). (**J**,**L**,**M**) Western blotting and quantitative data showing the protein expression of Cleaved caspases-3, BAX, BCL-2 (Ctrl: *n* = 4; other groups: *n* = 6). Glyceraldehyde-3-phosphate dehydrogenase (GAPDH) was used as loading controls for all Western blot assays. Data are expressed as means ± standard deviation (SD). * *p* < 0.05.

Although neoplasia involves many other processes, deregulated cell proliferation and decreased apoptosis have been proved to be a main contributor for neoplastic progression [22]. Given the high-proliferation effect of FGF1 and proapoptotic effect of RES, we utilized the CCK-8 assay and TUNEL staining to ascertain the therapeutic effect of RES to inhibit FGF1 mitogenic and proliferative activity in HepG2, 5637 and MCF-7 cells. The CCK-8 assay demonstrated that a slight rather than a significant decline of cell viability was detected in HepG2, 5637 and MCF-7 cells following RES treatment. Additionally, FGF1 markedly promoted proliferation at 100 ng/mL for 24 h, which was inhibited by RES (20 μM, 24 h) (Figure 1A–C). Suppressed cell death may be another driver for cell proliferation and provide the underlying platform for neoplastic progression. Thus, we subsequently explored the apoptosis by TUNEL staining in MCF-7 cells. The results showed that the FGF1 treatment suppressed the rate of TUNEL-positive cells, which was markedly reversed by RES (Figure 1D). Taken together, RES could significantly resist FGF1-induced cancer cell proliferation, suggesting RES may be a nutritional replenishment as auxiliary therapy in the chemotherapeutic treatment of different types of cancer.

### 3.2. RES and FGF1 Alleviated Myocardial Injury and Apoptosis in DOX-Related Mice without Tumor Promoting

Consistent with the above results in vitro, we found that the co-treatment of FGF1 and RES does not exert the morphologically developmental abnormalities in organs such as the heart, liver, kidney and testis (Figure S1). Therefore, to further examine the beneficial effects of RES/FGF1 co-administration in a DOX-induced cardiotoxicity, CK, LDH and cTnI were measured in serum 24 h after the injection of the DOX. The levels of CK, LDH and cTnI in the DOX-treated heart were obviously increased in comparison with the control (Ctrl) group. RES or FGF1 alone significantly reduced these changes. Meanwhile, a combination treatment with RES and FGF1 showed a synergistic effect in reducing myocardial damage than RES or FGF1 alone (Figure 1E–G). Additionally, as shown in Figure 1H, in comparison with the Ctrl group, the DOX obviously increased the TUNEL positive cells, which could be reversed by RES or FGF1. More importantly, the co-treatment of RES and FGF1 could further reduce cardiomyocyte apoptosis. Furthermore, the apoptosis-related markers, BCL-2, BAX and Cleaved caspase-3, which represent the final step for cell death, were also determined. Consistent with the TUNEL data, RES and FGF1 synergistically decreased the protein levels of Cleaved caspase-3 and increased the BCL-2/BAX ratio to sustain cardiomyocyte survival under DOX stress (Figure 1I–M).

### 3.3. RES and FGF1 Mitigated DOX-Induced Myocardial Inflammation in Mice

**Figure 2:**
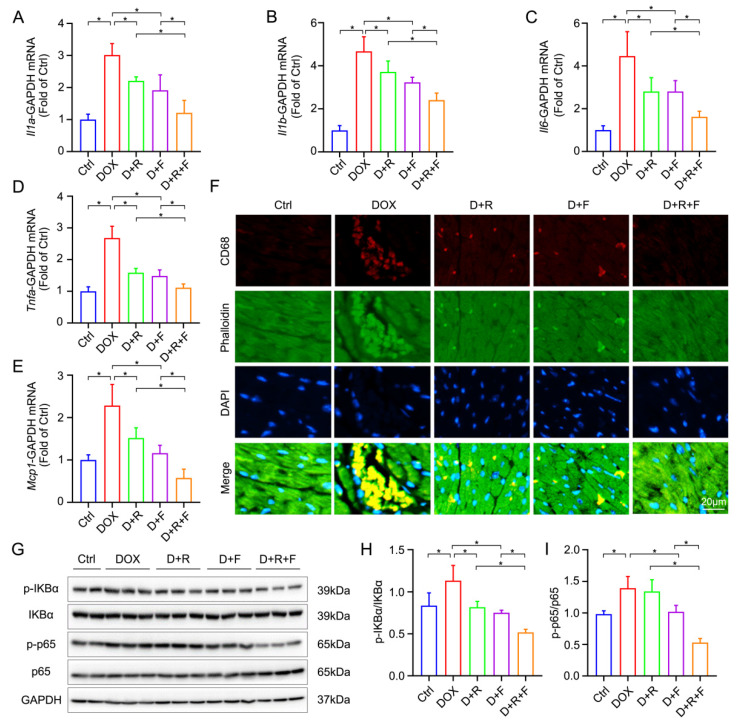
RES and FGF1 co-treatment mitigated DOX-induced myocardial inflammation in mice. (**A**–**E**) The relative mRNA levels of *Il1a*, *Il1b*, *Il6*, *Tnfa* and *Mcp1* were examined by qRT-PCR (*n* = 6). (**F**) Representative staining of CD68^+^ cell infiltrates (red). (**G**–**I**) The protein expression of p-IKBα, IKBα, p-p65, and p65 detected by Western blot analysis (Ctrl: *n* = 4; other groups: *n* = 6). GAPDH was used as loading controls for all Western blot assays. Data are expressed as means ± SD. * *p* < 0.05.

Substantial evidence has demonstrated that several pro-inflammatory cytokines in the myocardium largely contribute to DOX-induced cardiotoxicity [23,24]. Therefore, in the following study, myocardial inflammation was illustrated by the mRNA levels of interleukin-1α (*Il1a*), interleukin-1β (*Il1b*), interleukin-6 (*Il6*), tumor necrosis factor-α (*Tnfa*) and monocyte chemoattractant protein-1 (*Mcp1*). As shown in Figure 2A–E, in comparison with the Ctrl group, the DOX significantly elevated above pro-inflammatory cytokines mRNA levels, whereas the RES/FGF1 could obviously prevent DOX-associated inflammation. Additionally, the elevation of the DOX-related CD68^+^ macrophage infiltrates was further blunted via the combined treatment of RES and FGF1 (Figure 2F). The transcription factor NF-κB controls multiple aspects of innate and adaptive immune functions, which has also been implicated in heart tissue and regarded as a crucial mediator of inflammatory responses. Thus, IKBα and p65, inflammation-associated cytokines, in the cardiac tissue was detected via Western blotting. As expected, the phosphorylation of IKBα and p65 was dramatically increased by DOX, and the changes were noticeably reversed after the co-administration of RES and FGF1, compared with RES or FGF1 alone (Figure 2G–I).

### 3.4. RES and FGF1 Attenuated Oxidative Stress in DOX-Impaired Heart

**Figure 3:**
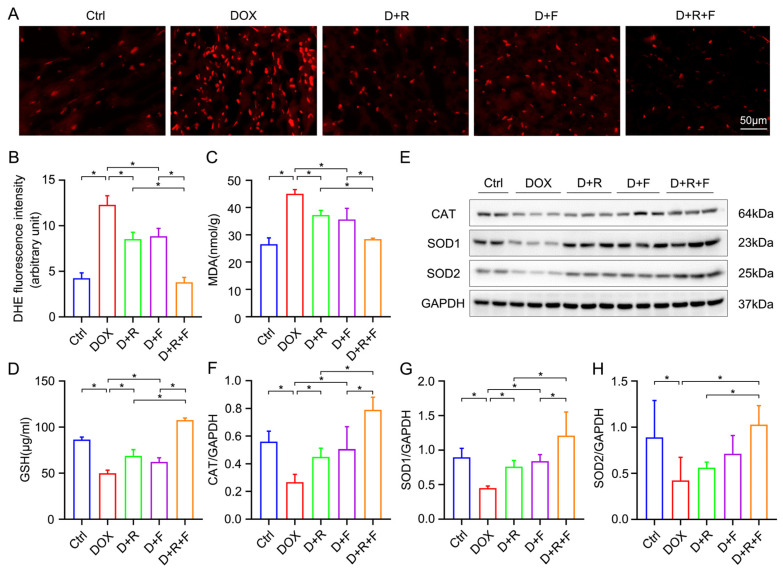
RES and FGF1 co-treatment attenuated oxidative stress in DOX-treated heart. (**A**,**B**) The levels of superoxide anion were detected by DHE staining (red) in cardiac tissues and quantification of fluorescence intensity (*n* = 3). (**C**) Quantitative results of MDA levels in cardiac tissues (*n* = 6). (**D**) The levels of GSH in serum (*n* = 6). (**E**–**H**) Quantification of cardiac CAT, SOD1 and SOD2 by Western blot analysis (Ctrl: *n* = 4; other groups: *n* = 6). GAPDH was used as loading controls for all Western blot assays. Data are expressed as means ± SD. * *p* < 0.05.

Generally, oxidative stress emerges as an essential role in DOX-induced acute cardiac damage. Herein, in order to assess the potential protective effect of RES, FGF1 and the co-treatment of them in DOX-induced cardiotoxicity, the levels of cellular ROS were detected by DHE staining. The ROS production was mitigated by RES or FGF1 in DOX-treated mice, and further alleviated in the co-treatment group (Figure 3A,B). In addition, DOX led to a decrease in the intracellular antioxidant capacity, which could be reversed by a co-treatment of RES and FGF1. There was an apparent increase in MDA and a significant reduction in GSH levels under DOX stress in comparison with the Ctrl group (Figure 3C,D). These effects were further corrected by the RES and FGF1 co-treatment in heart tissue. Additionally, antioxidant enzymes were implicated in ROS-scavenging force and pivotal for DOX tolerance in cardiomyocytes. The results in Figure 3E–H indicate that the expression of the three main antioxidant enzymes such as CAT, SOD1 and SOD2 were decreased in DOX-impaired cardiac tissue, however, this decrease was markedly rescued by the co-treatment of RES and FGF1 compared with either RES or FGF1.

### 3.5. Combination Treatment of RES and FGF1 Elevated the Activity of NRF2 in DOX-Induced Cardiotoxicity

**Figure 4:**
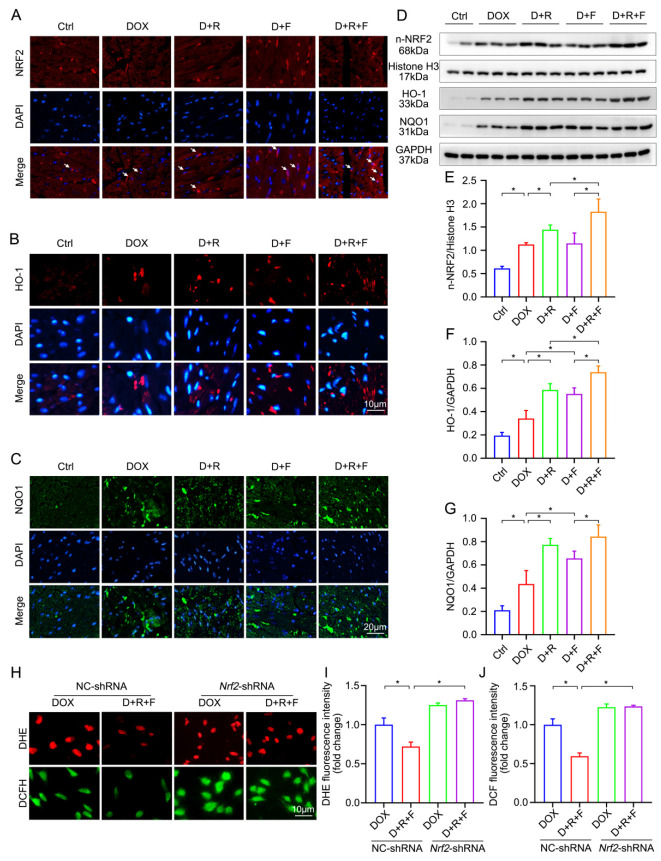
The activation of NRF2 was elevated after RES and FGF1 co-treating in DOX-treated mice. (**A**–**C**) The expression of NRF2, HO-1 and NQO1 was detected by immunofluorescent staining in cardiac tissues, respectively. (**D**–**G**) The levels of relative protein expression were examined by Western blot assay with densitometric quantification (Ctrl: *n* = 4; other groups: *n* = 6). (**H**–**J**) Representative images of DHE (red) and DCFH-DA (green) staining and quantification of fluorescence intensity in H9c2 cells (*n* = 3). GAPDH or Histone H3 was used as loading controls for all Western blot assays. Data are expressed as means ± SD. * *p* < 0.05.

NRF2, as a redox-sensitive transcription factor, could translocate into the nucleus where it induces the expression of downstream target genes such as *Ho-1* and *Nqo1* [25,26]. To verify whether the NRF2 antioxidant pathway played a crucial role in the protective actions of RES and FGF1, firstly, not only the nuclear translocation of NRF2, but also the activation of HO-1 and NQO1, the downstream enzymes of NRF2, were detected in heart tissue by IF staining and Western blot assay. The results have shown that the increase of nuclear NRF2 along with HO-1 and NQO1 in the DOX group were further augmented in a combined treatment of the RES and FGF1 group (Figure 4A–G). To confirm the critical role of NRF2 in RES/FGF1 co-treatment, we performed a knockdown of *Nrf2* with *Nrf2*-shRNA. NC-shRNA did not change the NRF2 expression and was regarded as a control vector. The knockdown of *Nrf2* significantly abolished most of the antioxidative capacity we had seen in Figure 4H–J.

### 3.6. Combination of RES and FGF1 Elevated the Activity of NRF2 via SIRT1

**Figure 5:**
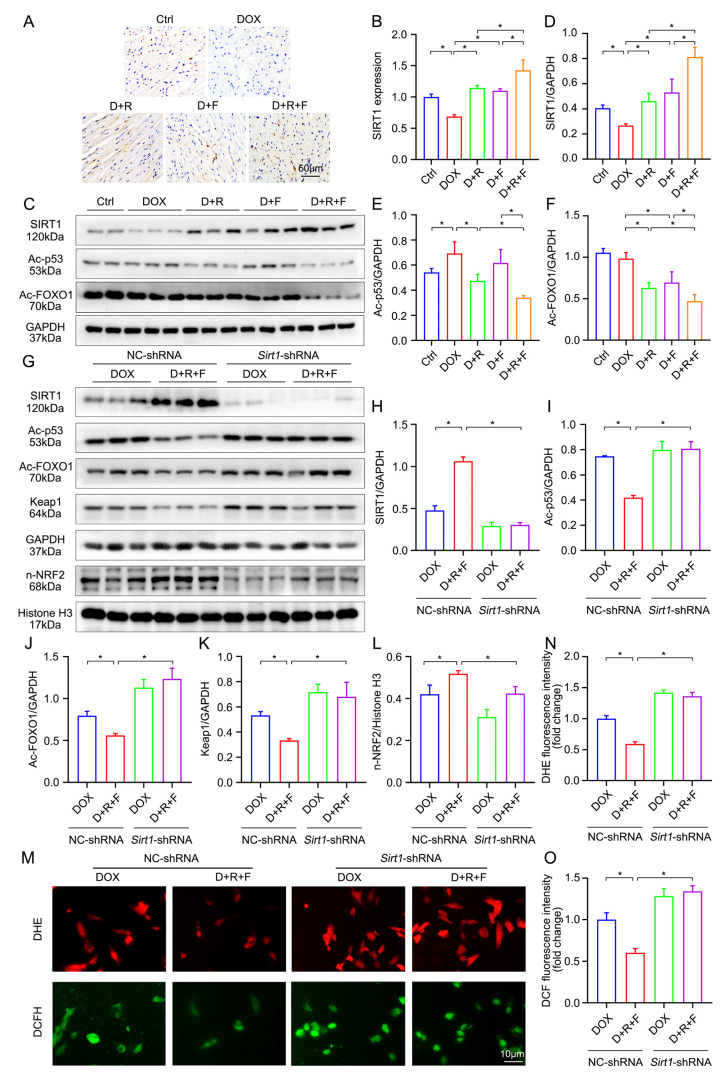
The combination of RES and FGF1 elevated the activity of NRF2 via SIRT1 in DOX-induced cardiotoxicity. (**A**,**B**) The expression of SIRT1 was supported by IHC staining (*n* = 6). (**C**–**F**) Cardiac protein abundance of SIRT1, Ac-p53 and Ac-FOXO1 as well as statistical results (Ctrl: *n* = 4; other groups: *n* = 6). (**G**–**L**) The expression of SIRT1, Ac-p53, Ac-FOXO1, Keap1 and NRF2 was detected by Western blotting (*n* = 3). (**M**–**O**) Representative images of DHE (red) and DCFH-DA (green) staining and quantification of fluorescence intensity in H9c2 cells (*n* = 3). GAPDH or Histone H3 was used as loading controls for all Western blot assays. Data are expressed as means ± SD. * *p* < 0.05.

Previous study has shown that RES activates SIRT1 to inhibit ROS generation in cardiomyocytes [27], and also the activation of SIRT1 can improve cardiac function by blocking the development of cardiac fibrosis in a model of DOX-induced cardiomyopathy [28]. Therefore, to verify whether the combination of RES and FGF1 can enhance the activity of SIRT1 in our model, the SIRT1 expression was evaluated by IHC staining. As shown in Figure 5A,B, there were significant reductions of SIRT1 in DOX-treated cardiac tissues and marked activation in the combined treatment of RES and FGF1 under the DOX stress. SIRT1 deacetylates numerous nonhistone protein substrates such as p53 and FOXO1, which is crucial in preventing cell stress. Based on this consideration, we quantified the Ac-p53 and Ac-FOXO1 protein expression by Western blotting in vivo; the result indicated that a combination of RES and FGF1 significantly upregulated the deacetylation of p53 and FOXO1 after the DOX treatment (Figure 5C–F).

To address whether NRF2 affects cell protective responses to RES/FGF1 via SIRT1, we performed a knockdown of *Sirt1* with *Sirt1*-shRNA. NC-shRNA did not affect SIRT1 expression, and in subsequent studies it was used as a control vector. Firstly, the knockdown of the *Sirt1* expression blocked most of the protective effects we had seen with the RES and FGF1 treatment. Importantly, as shown in Figure 5G–L, *Sirt1*-shRNA eliminated the effect of RES in combination with FGF1 on promoting the nuclear accumulation of NRF2 as well as downregulating the protein expression of Ac-p53, Ac-FOXO1 and Keap1. Moreover, the protective effect of RES/FGF1 co-treatment on oxidative stress detected by DHE and DCFH-DA staining was also concomitantly diminished in *Sirt1* knockdown cells (Figure 5M–O).

### 3.7. Crosstalk between SIRT1 and NRF2 Shapes Cytoprotective Responses

**Figure 6:**
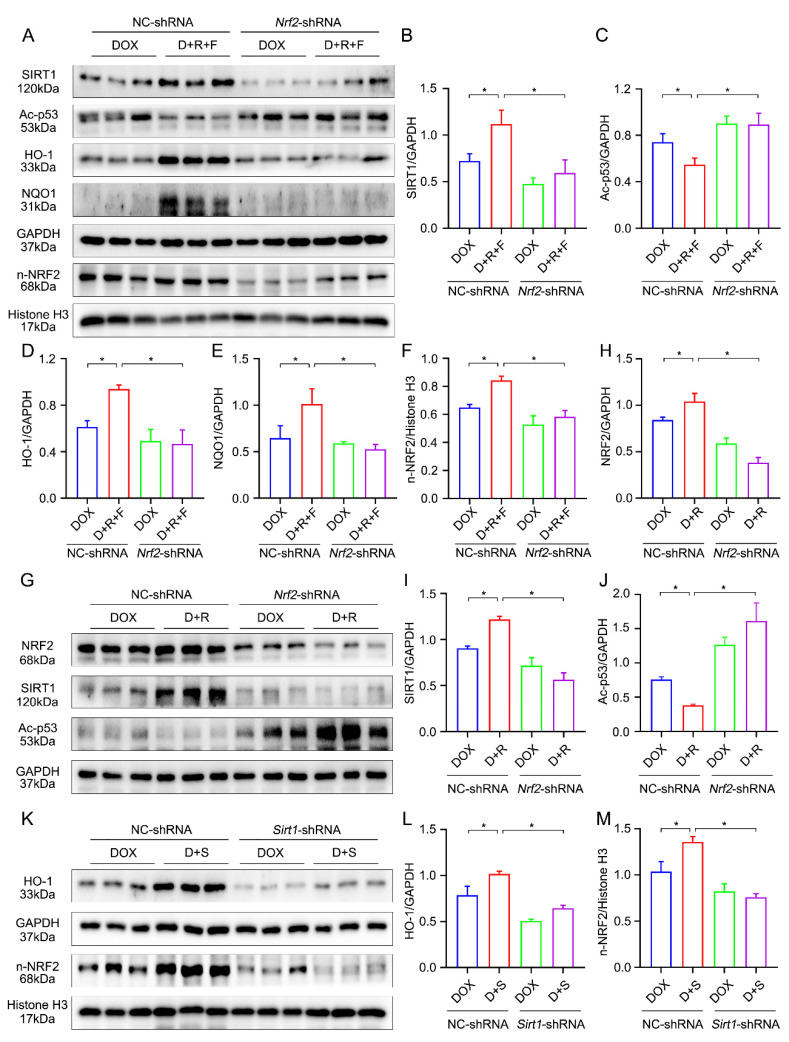
The interaction between SIRT1 and NRF2 in vitro. (**A**–**F**) The expression of SIRT1, Ac-p53, HO-1, NQO1 and NRF2 was detected by Western blotting after transfection with NC-shRNA or *Nrf2*-shRNA in H9c2 cells of each group (*n* = 3). (**G**–**J**) The protein expression of NRF2, SIRT1 and Ac-p53 were examined by Western blot assay with densitometric quantification transfection with NC-shRNA or *Nrf2*-shRNA in H9c2 cells of different groups (*n* = 3). (**K**–**M**) The expression of HO-1 and NRF2 was detected by Western blotting and quantified by densitometry after transfection with NC-shRNA or *Sirt1*-shRNA in H9c2 cells of different groups (*n* = 3). GAPDH or Histone H3 was used as loading controls for all Western blot assays. Data are expressed as means ± SD. * *p* < 0.05.

To further determine the relationship between SIRT1 and NRF2, *Nrf2*-shRNA was transfected in our cell model. Interestingly, both SIRT1 protein abundance and NRF2 downstream gene activation were also significantly decreased, which accompanied the ROS production and imbalanced redox homeostasis after the *Nrf2* knockdown (Figure 4H–J and Figure 6A–F). The above findings all demonstrate that RES and FGF1 have a positive effect on the activation of the NRF2 and SIRT1 pathway. We further postulated whether there is a positive feedback regulation between SIRT1 and NRF2. Interestingly, the results of Figure 6G–J demonstrated that the shRNA-mediated silencing of *Nrf2* obviously reduced the protein expression and activity of SIRT1, and blocked the RES-induced activation of SIRT1. Moreover, by using a well-characterized NRF2 activator (SFN) [29], we further found that the protein expression of NRF2 and HO-1 were significantly increased, but this effect was largely cancelled by *Sirt1*-shRNA (Figure 6K–M). The above results demonstrate that SIRT1 and NRF2 might crosstalk and form a positive feedback loop to perform the protective effect on the heart in response to DOX.

## 4. Discussion

DOX, a kind of widely used anthracycline antibiotic, has attracted much attention for its powerful anti-tumor capacity, whereas its serious side effect on cardiotoxicity limits its clinical application [30,31]. Thus, a promising pharmacological therapy for DOX-induced cardiotoxicity is urgently needed to be addressed. The present study provides three new lines of evidence implicating the co-treatment of RES with FGF1 therapy in DOX cardiotoxicity: the first novel finding is that RES could antagonize the proliferative activities and potential tumorigenic risks of FGF1. The second new finding is that the anti-oxidant, anti-inflammation and anti-apoptosis benefit of RES combined with FGF1 in heart requires the activation of NRF2 antioxidant signaling. Our third innovative finding is that a positive autoregulatory feedback loop between SIRT1 and NRF2 mediates an RES- and FGF1-induced NRF2 activation. Either *Sirt1* deletion or *Nrf2* inhibition with genetic approaches abrogated RES/FGF1-mediated therapeutic effects on DOX-induced cardiotoxicity.

Our study and other previous studies have verified that FGF1, as a powerful metabolic hormone, played a pivotal role in diabetic complication and displayed the favorable effects on maintaining myocardial integrity and protecting against cardiac dysfunction in response to DOX [8,32,33,34,35]. Therefore, FGF1 has a great prospect of clinical application in cardiovascular disease. However, the long-term application of wild-type FGF1 may enhance tumorigenic risks owing to its strong mitogenic activity, especially because FGF1’s proliferative activities may possibly interfere with the effect of chemotherapeutic drugs. In this study, we determined that RES significantly decreased FGF1-induced cancer cell proliferation as well as unregulated apoptosis, suggesting RES, as an adjuvant drug, could effectively avoid the side effects of FGF1 application, especially in cancer-prone disease.

Another new finding of this study is the favorable effects of the co-treatment of RES and FGF1 on the myocardium that requires the activation of the NRF2 antioxidant signaling. NRF2 has been implicated in regulating a long list of antioxidative genes’ expression to resist oxidative stress. Previous studies have demonstrated that Keap1 binds with NRF2 in cytoplasm and promotes its ubiquitination and degradation [36]. When oxidative stress occurs, NRF2 detaches from Keap1 and translocates to the nucleus, where it heterodimerizes with one of the small Maf proteins and further associates with antioxidant response elements (AREs) to resist the stress [37,38]. In this study, we found that RES in combination with FGF1 could markedly increase the nuclear accumulation of NRF2 and upregulates the expression of downstream antioxidant target genes, which further ameliorates DOX-induced myocardial injury, apoptosis, inflammation and oxidative stress. Nevertheless, the cardioprotective effects of RES in combination with FGF1 were abolished when *Nrf2* was silenced. This suggested that the ability of the co-treatment of RES and FGF1 was dependent on NRF2 antioxidant signaling.

Our third important new finding of this study is that SIRT1 is an essential contributor for cardio-protective effects following RES and FGF1 treatment, which form a positive feedback loop between SIRT1 and NRF2 in improving DOX-induced heart injury. Emerging evidence indicates that the crucial role of SIRT1 has attracted extensive attention in cardiovascular disease with a critical ability of SIRT1 to resist DOX-induced oxidative damage and cell death [39]. FOXO1 is a direct target of SIRT1 deacetylation, which could in turn induce its transcriptional activity on both SIRT1 and ROS scavenger promoters [40]. A recent study by Zhao et al. demonstrated that p53 acetylation and its cytoplasmic localization were dramatically decreased with the overexpression of SIRT1, which was accompanied with attenuated cells apoptosis [41]. In the current study, we elucidated the deacetylation of p53 and both this and FOXO1 were indeed further increased after the combined treatment of RES with FGF1, suggesting that the activation of SIRT1 was more distinctly enhanced after the RES/FGF1 co-treatment. Moreover, an in vitro study on murine microglia exhibited that melatonin inhibited the NLR Family Pyrin Domain Containing 3 (NLRP3) inflammasome activation and pyroptosis, which was substantially dependent on the crosstalk between NRF2 and SIRT1 [42]. Another study by Huang et al. reported that the feedback loop between the SIRT1 and Keap1/NRF2/ARE anti-oxidative pathway inhibit the protein expressions of fibronectin and the transforming growth factor-β1 in AGEs-treated glomerular mesangial cells [43]. In our current study, *Sirt1*-shRNA blocked the RES in combination with the FGF1 that induced the NRF2 upregulation as well as antioxidant function activation. However, when *Nrf2* was inhibited, the expression of SIRT1 and its antioxidative effects on the cardiomyocyte declined, indicating that there is a positive feedback effect between SIRT1 and NRF2 in response to the DOX injury, which in turn inhibits DOX-induced cardiotoxicity.

In summary, our findings demonstrate that RES can not only inhibit the proliferative activity of FGF1, but also enhance the antioxidant effect of FGF1 to protect against DOX-induced cardiotoxicity, which is predominantly owing to a positive autoregulatory feedback loop between SIRT1 and NRF2. These findings suggest that a combined treatment with RES and FGF1 could be taken into consideration as a potential therapeutic target of anthracycline cardiotoxicity in a cancer population during chemotherapy.

## Data Availability

Not applicable.

## Supplementary Materials

The following supporting information can be downloaded at: https://www.mdpi.com/article/10.3390/nu14194017/s1, Figure S1: Representative different organs images.
