# Supplementary figures and images for: Resveratrol and FGF1 Synergistically Ameliorates Doxorubicin-Induced Cardiotoxicity via Activation of SIRT1-NRF2 Pathway
Source: Nutrients. 2022 Sep 27;14(19):4017. doi: 10.3390/nu14194017 (PMC9572068; doi:10.3390/nu14194017)

## Supplementary Materials

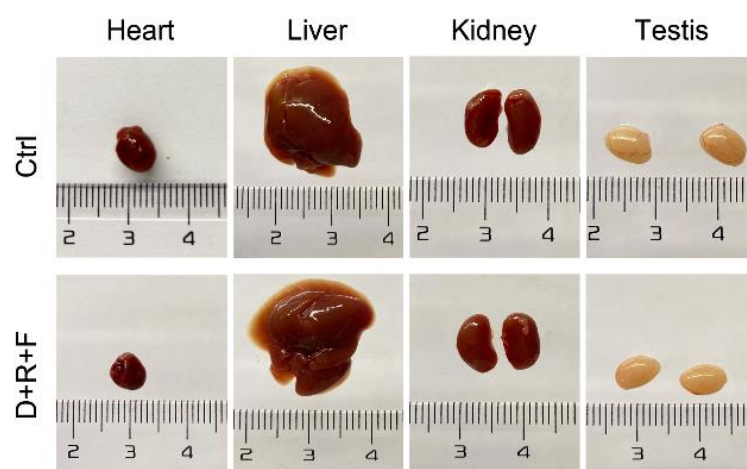

**Figure S1.** Representative different organs images.

Supplement: Supplementary file 1 [file nutrients-14-04017-s001.zip › nutrients-1873778-supplementary.pdf]
